# Xeroderma pigmentosum associated with recurrent squamous and basal cell carcinoma: a case report from Nepal

**DOI:** 10.1097/MS9.0000000000003804

**Published:** 2025-09-05

**Authors:** Prayash Paudel, Neetika Paudel, Ram Aryal, Sajish Khadgi, Rashmita Acharya, Midori Pote Shrestha, Aashish Bhandari, Pramath Kapoor

**Affiliations:** a Bachelor of Medicine and Bachelor of Surgery, Maharajgunj Medical Campus, Tribhuvan University Teaching Hospital, Institute of Medicine, Maharajgunj, Kathmandu, Nepal; b Department of ENT-Head and Neck Surgery, Maharajgunj Medical Campus, Tribhuvan University Teaching Hospital, Institute of Medicine, Maharajgunj, Kathmandu, Nepal; c Department of Pathology, Maharajgunj Medical Campus, Tribhuvan University Teaching Hospital, Institute of Medicine, Maharajgunj, Kathmandu, Nepal

**Keywords:** DNA repair, melanoma, squamous cell carcinoma, xeroderma pigmentosum

## Abstract

**Introduction:**

Xeroderma pigmentosum (XP) significantly increases the risk of skin malignancies due to impaired DNA repair mechanisms. The estimated incidence is 1 in 250 000 in the USA. Patients with XP are more likely to develop cutaneous neoplasms, primarily melanoma, squamous cell carcinoma (SCC), and basal cell carcinoma (BCC), and the most prevalent cutaneous and ocular tumor is SCC.

**Case presentation:**

We present a case of a 21-year-old male from western Nepal who presented to Tribhuvan University Teaching Hospital with multiple lesions on the face and was diagnosed with SCC and BCC. We comprehensively evaluated the chronology of events and associated pathology, and a detailed review of prior medical and surgical records was performed.

**Discussion:**

XP is an uncommon, autosomal recessive disorder with mutations in nucleotide excision repair genes. Patients with XP are more likely to develop cutaneous and ocular neoplasms early in life, which are more aggressive and have a high recurrence rate.

**Conclusion:**

Patients with XP can prolong their lives and avoid developing skin cancer by receiving early diagnosis and broad sun protection.

## Introduction

Xeroderma pigmentosum (XP, literally dry pigmented skin) is a rare, inherited autosomal recessive illness characterized by heightened sensitivity to ultraviolet (UV) radiation, leading to severe sunburn, alterations in skin pigmentation, and a significantly increased risk of skin malignancies due to impaired DNA repair mechanisms^[1–3]^. These patients exhibit a 1000-fold increased chance of acquiring early malignant neoplasms, predominantly skin malignancies^[4]^. XP impacts both males and females uniformly; nevertheless, the prevalence of the illness differs across nations. Japan has an estimated incidence of 1 in 20 000 healthy children and is the country with the highest incidence, while it has an estimated incidence of 1 in 250 000 in the USA. Approximately 2.3 per million live births are diagnosed with XP in Western Europe^[5]^.

**HIGHLIGHTS**

- Xeroderma pigmentosum (XP) is an autosomal recessive disorder with mutations in nucleotide excision repair genes.
- Two of the most common malignancies in XP patients are basal cell carcinoma and squamous cell carcinoma .
- To comprehensively evaluate the chronology of events and associated pathology, a detailed review of prior medical/surgical records and histopathological reports is performed.
- The case was managed with wide local excision of the lesions with histopathological margin assessment, followed by bilateral selective neck dissection for lymph node involvement and close surveillance for recurrence.
- The delayed diagnosis of XP in a 21-year-old patient, despite a long history of recurrent UV-induced skin malignancies and characteristic skin changes, reflects challenges encountered in resource-limited settings.
- A multidisciplinary approach involving dermatologists, surgeons, oncologists, and pathologists ensures comprehensive management and optimal patient outcomes.

The etiology may involve mutations in as many as eight genes: seven from XPA to XPG, which are important for nucleotide excision repair (NER) of DNA damage, and the eighth gene (XPV), which produces the variant subtype of the disease. The clinical manifestations differ on the basis of the disease subtypes; nonetheless, cutaneous lesions typically emerge early in development and are provoked by heightened photosensitivity. They start as severe sunburns, even with minimal exposure, and may progress to solar melanomas, followed by areas of hyper or hypopigmentation, premature aging, poikiloderma, and areas of high risk for neoplasms^[6]^. UV light induces the synthesis of photoproducts in DNA, which predisposes individuals to carcinogenesis and facilitates cellular aging, apoptosis, and mutagenesis^[7]^. Patients with XP are more likely to develop cutaneous neoplasms, primarily melanoma, squamous cell carcinoma (SCC), and basal cell carcinoma (BCC), and the most prevalent cutaneous and ocular tumor is SCC. The management of these malignancies is challenging due to their aggressive and multifocal nature. Surgical excision remains the mainstay of treatment; however, reconstructive challenges and the risk of recurrence require careful planning. In this context, the study by Bertozzi *et al*^[8]^ provides valuable single-center evidence supporting the effectiveness of surgical treatment for BCC of the head and neck, demonstrating high rates of complete tumor removal and low recurrence when adequate margins and appropriate reconstructive techniques are employed. Incorporating such evidence into XP management underscores the importance of meticulous surgical intervention tailored to preserve function and aesthetics, which is crucial given the frequent involvement of cosmetically sensitive facial areas in XP patients. Patients with XP can prolong their lives and avoid developing skin cancer by receiving early diagnosis and broad sun protection^[4]^.

Ocular surface squamous neoplasia (OSSN), coined by Lee and Hirst, encompasses the full range of dysplastic and carcinomatous lesions of the ocular surface; conjunctival and corneal intraepithelial neoplasms were later added. Recurrence rates for preinvasive and invasive OSSN (when dysplastic cells penetrate the basement membrane, it is known as invasive carcinoma) after surgical excision vary from 15% to 52%^[9]^. Surgery (wide local excision) and medication (0.04% mitomycin, interferon alpha 2b, 1% 5-fluorouracil) are available forms of treatment. Both interferon and mitomycin are equally successful in resolving OSSN, according to comparative research^[10]^.

We present a case of a 21-year-old male who presented to our center with multiple lesions on the face and was diagnosed with SCC and BCC over xeroderma pigmentosa in accordance to the SCARE guideline^[11]^.

## Case presentation

**Figure 1.**
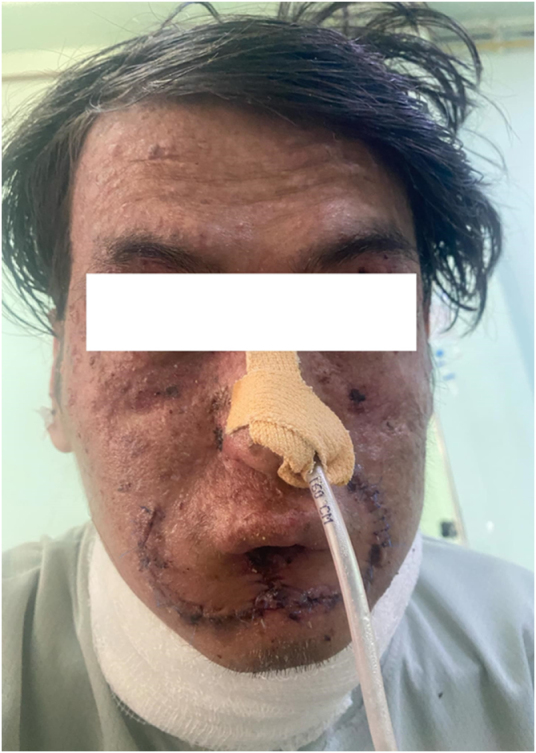
Clinical presentation of patient.

A 21-year-old nonsmoker, nonalcohol consumer male from a rural hilly region of western Nepal presented to our center with a progressively enlarging and painful ulcer on the lower lip. The ulcer had been present for several months, measured approximately 2 × 1 cm, and was tender to touch, with no spontaneous bleeding reported. Additionally, the patient exhibited a salt-and-pepper pattern with hyperpigmented macules predominantly on sun-exposed areas, as shown in Figure 1, including the face and neck. On examination, a 0.5 × 0.5 cm hard, slightly mobile swelling was noted in the right submandibular region, suggesting possible lymphadenopathy. The patient’s medical history revealed a long-standing pattern of recurrent skin lesions, many of which were surgically treated at various times in different healthcare facilities.

To comprehensively evaluate the chronology of events and associated pathology, a detailed review of prior medical/surgical records and histopathological reports was performed, and the results are summarized in Table 1.

**Table 1:** Summary of prior medical/surgical records and histopathological findings of patient

Date	Site	Diagnosis	Tumor characteristics	Treatment
15 June 2023	Left cheek	Keratotic BCC	Thickness: 3.7 mm, Clark IV, mitotic: 0–2/HPF, pT1 staging (N not assigned)	
	Left ala of nose	BCC	Peripheral palisading, dysplastic epithelium	
	Dorsum of nose	SCC *in situ*	Loss of polarity, hyperchromatic nuclei	
30 June 2023	Left lower eyelid	Bowenoid actinic keratosis (KIN II)	Acanthosis, hyperchromatic nuclei	
24 October 2023	Upper lip	Favors keratoacanthoma	Minimal atypia, lymphoid infiltration	Excision of lesions over nasolabial fold under LA, specimen sent for HPE
	Nasolabial fold	BCC	Infiltrating nests, retraction artifact	
16 December 2023	Left lower eyelid	Well-differentiated SCC	Keratin pearls, mitotic: 1–2/HPF	Left-lower lid mass excision with lid reconstruction was done on 25 December 2023.
11 April 2024	Right cheek	Well-differentiated SCC	Thickness: 2.1 mm, Clark III, mitotic: 2–5/HPF, pT1 staging	
	Right eyebrow	Moderately differentiated SCC	No keratin pearls, 0–2/HPF	
	Nose	Moderately differentiated SCC	Thickness: 2.1 mm, Clark IV, 0–2/HPF, pT1 staging	
24 April 2024	Lower lip	SCC *in situ* with suspicious invasion	Full-thickness dysplasia	Wide local excision of the lower lip lesion along with bilateral selective neck dissection (Level IIA, IIB, and III nodes along with fibrofatty tissue) and local flap reconstruction (Karapandzic flap) under general anesthesia.
7 July 2024	Right nasolabial fold and lower lip	SCC	FNAC: reactive lymphadenopathy	
28 July 2024	Right temple	Well-differentiated SCC	Thickness: 2.5 mm, Clark III, 0–1/HPF, pT1 staging	Excision of lesions over the right temple, left cheek, under local anesthesia. Samples were sent for HPE.
	Left cheek	Basosquamous carcinoma	Thickness: 2.5 mm, Clark IV, 0–2/HPF, pT1 staging	
27 July 2024	Right temple, left cheek, right ala	Lesion excision	-	
10 August 2024	CT neck	Right lower lip lesion; R IIb LN (13 × 6.4 mm)	Suspicious of metastasis	

The details in chronological order are presented hereby:

15 June 2023

**Figure 2.**
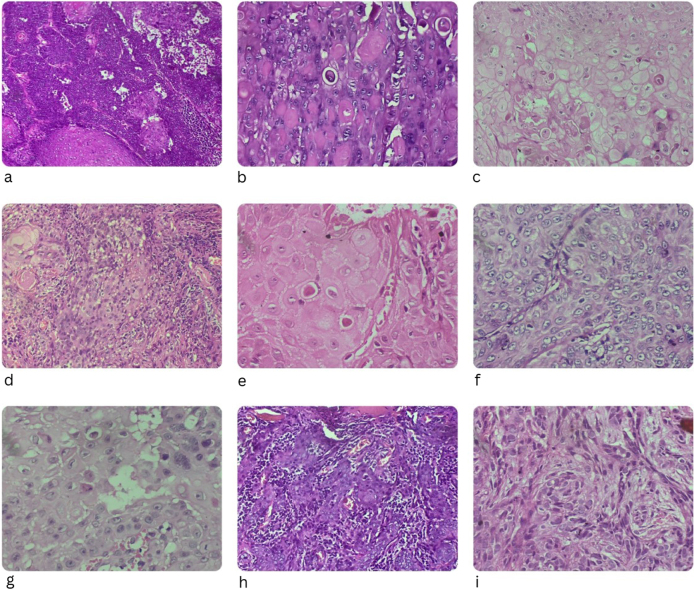
Histopathological images.

The patient presented with multiple raised papules with ulceration over the left lateral nasal bridge, right nasal ala, and left malar region. Excisional biopsy of the skin from the cheek and nose was performed, which revealed the following:

1. The section examined from the cheek shows nests and cords of basaloid cells infiltrating the full thickness of the dermis, with the tumor thickness measuring 3.7 mm. These nests show peripheral palisading and retraction artifacts. Most of these nests show squamous differentiation, with some showing keratin pearls. The number of mitotic figures ranged from 0 to 2/high-power field (HPF). Lymphovascular and perineural invasion were not observed. The subcutis is not present in the section examined. The tumor extends to the edges as well as the deep margin of the section examined. The histopathology image is shown in Figure 2a.
2. The section examined from the left ala of the nose shows infiltrating cords and nests of basaloid cells. These nests show peripheral palisading and retraction artifacts. The overlying squamous epithelium is focally dysplastic. The histopathology image is shown in Figure 2b.
3. A section from the dorsum of the nose shows the acanthotic epidermis and underlying dermis. The epidermis shows a loss of polarity involving nearly full thickness. The nuclei were hyperchromatic, and mitotic figures were also observed. The tumor did not extend into the edges of the section examined.

Diagnosis:

1. Skin, cheek: excisional biopsy:
  - Keratotic BCC.
  - Maximum tumor dimension: 1.0 cm.
  - Tumor thickness: 3.7 mm.
  - Clark level: IV.
  - Lymphatic, vascular, and perineural invasions: Not observed.
  - Cut margins: The tumor extends into the edges of the section examined and deep margins.
  - TNM (AJCC) stage: pT1N not assigned.
2. Skin, ala of the nose, left: incisional biopsy: BCC.
3. Skin, dorsum of the nose: excisional biopsy:
  - SCC *in situ*.
  - The tumor did not extend into the edges of the section examined.

30 June 2023

1. For a mass with tenderness and hypopigmentation present on the left lower eyelid for 3 months (D/D: sebaceous gland carcinoma, BCC), an incisional biopsy of the skin from the left lower eyelid was performed. The section shows an acanthotic epidermis with loss of polarity involving the lower half. The nuclei are hyperchromatic. Parakeratosis is also observed. No dermal tissue is observed in the section examined. The diagnosis was moderate keratinocytic intraepithelial neoplasia (Bowenoid actinic keratosis). The histopathology image is shown in Figure 2c.

24 October 2023

Biopsy of the upper lip and nasolabial fold revealed the following:

1. The section examined from the “Upper lip” shows a keratin-containing crater with peripheral lipping of the epidermis. The epidermis is acanthotic with downward proliferation. Cytological atypia is minimal, with occasional mitotic figures. The superficial dermis showed moderate interstitial lymphocytic infiltration.

4. Sections examined from the “Nasolabial fold” show nests and cords of basaloid cells infiltrating the dermis. Retraction artifacts are observed in a few nests. These cells have oval hyperchromatic nuclei with occasional mitotic features. Continuity of the tumor with the overlying epidermis was observed. The histopathology image is shown in Figure 2d.

Diagnosis:

- BCC of the nasolabial fold.
- The architectural pattern favors keratoacanthoma. Excisional biopsy is advised.

16 December 2023

1. Biopsy of skin from the left lower eyelid: the section shows infiltrating tumor cells arranged in compact nests and lobules. The tumor cells have abundant eosinophilic cytoplasm, round to oval nuclei, vesicular chromatin, and prominent nucleoli. Keratin pearls and intercellular bridges are observed. Mitotic figures constitute 1–2/HPF in mitotically active areas. The histopathology image is shown in Figure 2e.

The patient was diagnosed with well-differentiated SCC.

25 December 2023

A left lower lid mass excision with lid reconstruction was performed.

11 April 2024

After 4 months, the patient presented with a skin-colored nodule over the right cheek and dorsum of the nose. Excisional biopsy of the skin from the right cheek, right eyebrow, and nose was performed to rule out BCC or SCC.

1. Sections from the right cheek biopsy revealed an acanthotic epidermis with elongation of rete ridges and parakeratosis. Focally, the epidermis shows full-thickness dysplasia. There are nests of atypical squamoid cells infiltrating the papillary dermis, with a tumor thickness of 2.1 mm. The reticular dermis and subcutis are free of the tumor. These nests show stratification, keratin pearls, and moderate cellular pleomorphism. Mitotic figures constitute 2–5/HPF in mitotically active areas. Lymphatic, vascular, and perineural invasions are not found. The tumor was 2.3 mm away from the deep margin. All cut margins were free of the tumor.
2. Sections from the right eyebrow biopsy revealed multiple fragments of the epidermis with underlying dermis. One of the fragments shows nests and lobules of atypical squamoid cells infiltrating the dermis. Keratin pearls are not found. These cells show moderate cellular pleomorphism, with mitotic figures constituting 0–2/HPF in mitotically active areas. Lymphatic, vascular, and perineural invasions are not found.

5. Sections from the nose biopsy sample showed nests of atypical squamoid cells infiltrating the papillary and reticular dermis, with a tumor thickness of 2.1 mm. These nests show stratification and moderate cellular pleomorphism. Mitotic figures constitute 0–2/HPF in mitotically active areas. Keratin pearls are not observed. The tumor was microscopically measured as 4.5 mm. All cut margins were free of the tumor. The histopathology image is shown in Figure 2f.

Diagnosis:

1. Skin, cheek, right: excisional biopsy:
  - Well-differentiated SCC.
  - Maximum tumor dimension: 9.0 mm.
  - Tumor thickness: 2.1 mm.
  - Clark level: III.
  - Lymphatic, vascular, and perineural invasions: Not observed.
  - All cut margins were free of the tumor.
  - TNM classification (AJCC 8th edition): pT1 N not assigned.
2. Skin, eyebrow, right: moderately differentiated SCC.
3. Skin, nose: moderately differentiated SCC.
  - Maximum tumor dimension: 4.5 mm (microscopically measured).
  - Tumor thickness: 2.1 mm.
  - Clark level: IV.
  - Lymphatic, vascular, and perineural invasions: Not observed.
  - All cut margins were free of the tumor.
  - TNM classification (AJCC 8th edition): pT1 N not assigned.

24 April 2024

- A single erythematous scaly plaque was present over the lower lip.
- An incisional biopsy of the skin from the lower lip was performed, with a differential diagnosis of SCC.
- Microscopic findings from the biopsy:
  - The sections revealed a focally hyperplastic epidermis with an underlying dermis.
  - The epidermis demonstrated nearly full-thickness dysplasia with downward growth into the dermis.
  - The epidermal cells exhibited pleomorphism with coarse nuclear chromatin and prominent nucleoli.
  - Mitotic figures were present.
  - The intervening dermis displayed a dense infiltrate of lymphocytes and plasma cells.

The histopathology image is shown in Figure 2g.

Diagnosis:

- Skin, lip, lower: incisional biopsy findings suggest SCC *in situ* with suspicious invasion.
- Excisional biopsy was advised for confirmation.

7 July 2024

- The patient presented with multiple tumors, including SCC of the lower lip and right nasolabial fold.
- There was right submandibular lymphadenopathy. Fine-needle aspiration cytology (FNAC) of the lymph node was advised.

FNAC findings (right submandibular lymph node):

- Blood-mixed aspirate revealed a mixed population of lymphoid cells at various stages of maturation, tingible body macrophages, and a few histiocytes.
- No granulomas, necrosis, or atypical cells were identified.

Diagnosis:

- Reactive lymph nodes.

28 July 2024

- Excisional biopsy was performed for the following lesions in a known case of XP:
  - Ulcerated plaques over the right nasolabial fold, left cheek, and right earlobe.
  - A single skin-colored nodule over the right temple.

Histopathological findings:

1. Temple, right (well-differentiated SCC):
  - The sections revealed nests and lobules of atypical squamous cells infiltrating the superficial dermis with a tumor thickness of 2.5 mm.
  - The tumor showed stratification, keratin pearls, and intercellular bridges.
  - The tumor cells were moderately pleomorphic, with 0–1 mitotic figures per HPF.
  - No lymphatic, vascular, or perineural invasion was observed.
  - All cut margins were free of tumor. The nearest resection margin was the deep margin, which was 1.3 mm away.
  - Tumor dimensions: 6.0 mm.
  - Depth of invasion: into the papillary-reticular dermal interface (Clark level III).

The histopathology image is shown in Figure 2h.

2. Cheek, left (Basosquamous carcinoma):
  - The sections revealed nests, cords, and lobules of basaloid cells arising from the epidermis and extending into the full-thickness dermis with a tumor thickness of 2.5 mm. The subcutis was free of tumor.
  - The tumor showed peripheral palisading, retraction artifacts, and squamoid differentiation with keratin pearls.
  - There were up to 2 mitotic figures per HPF.
  - No lymphatic, vascular, or perineural invasion was observed.
  - All four resection margins were free of tumor. The nearest resection margin was the deep margin, 0.7 mm away.
  - Tumor dimensions: 4.0 mm.
  - Depth of invasion: full-thickness dermis (Clark Level IV).

The histopathology image is shown in Figure 2i.

Final diagnoses (AJCC 8th Edition Staging):

1. Right temple: well-differentiated SCC.
  - Tumor stage: pT1 (N not assigned).
2. Left cheek: basosquamous carcinoma.
  - Tumor stage: pT1 (N not assigned).

27 July 2024

- Under proper precautions, the lesions were excised from the right temple, left cheek, and right ala of the nose (nasolabial fold) under local anesthesia.

10 August 2024

- CT neck findings are as follows:
  - An approximately 2 × 1 × 1 cm heterogeneous enhancing soft tissue density was noted on the right side of the lower lip.
  - An approximately 13 × 6.4 mm enhancing lymph node was noted in the right IIb level, likely indicating malignant pathology with suspicion of nodal metastasis.

Most lesions occurred in sun-exposed areas and were frequently associated with malignancy. This pattern of recurrent skin malignancies in conjunction with photosensitivity raised a strong clinical suspicion of XP, a rare autosomal recessive disorder caused by defective NER mechanisms. XP predisposes individuals to UV-induced DNA damage, leading to early-onset skin malignancies.

Other investigations included hematology, biochemistry, immunology, parasitology, and urine sample registration, which yielded unremarkable results. The CT head and neck images revealed an approximately 2 × 1 × 1 cm heterogeneously enhanced soft tissue density noted on the right side of the lower lip, and an approximately 13 × 6.4 mm size enhancing lymph node noted on the right level IIb (likely malignant pathology with suspicion of nodal metastasis).

As mentioned in Table 1, the patient underwent a wide local excision with selective neck dissection and flap reconstruction. The postoperative course was uneventful, and the patient was discharged after 10 days with the advice of avoiding excessive exposure to sunlight, applying sunscreen frequently as per instructions, and being on regular follow-up visits in the ENT, plastic surgery, and ENT OPDs.

This case highlights the importance of early detection, prevention of sun exposure, and periodic monitoring for malignancies in XP patients, especially in regions with limited healthcare resources. The absence of a prior molecular diagnosis or family history of similar presentations reflects the challenges of diagnosing rare genetic disorders in resource-constrained settings. Furthermore, the repeated malignancies in this patient underscore the significant unmet need for early preventive interventions and genetic counseling in such cases. Given the rural setting of Nepal, the diagnosis of XP was delayed due to limited awareness and access to dermatological care.

## Discussion

We present the case of a 21-year-old male patient from a consanguineous family with multiple oculocutaneous malignancies at the time of presentation and a history of multiple surgeries for the recurrence of a neoplasm of the skin. He was not diagnosed with XP until a year ago at our center on the basis of the clinical presentation, even though he had some salt-and-pepper appearance of the sun-exposed parts and UV-exposure-related recurring SCCs and BCCs. In our country, very few cases have been reported, and a study by Karmacharya *et al* in 1987 revealed that most cases were from the western Kathmandu and Lumbini zones^[12]^.

The delayed diagnosis of XP in this 21-year-old patient, despite a long history of recurrent UV-induced skin malignancies and characteristic skin changes, reflects several challenges commonly encountered in resource-limited settings like Nepal. First, the rarity of XP and limited awareness among healthcare providers contribute to missed or late diagnosis. Many patients present only after developing multiple malignancies or severe phenotypic features, as early subtle signs such as lentiginosis or mild photosensitivity may be overlooked or attributed to common sun damage. Second, the lack of specialized diagnostic facilities, including genetic testing and DNA repair assays, hinders early confirmation of XP, as these advanced tools are often unavailable or inaccessible in Nepal. Third, social and cultural factors, including consanguineous marriages and limited health education, may delay presentation and recognition of inherited disorders. Studies from Nepal and neighboring regions have reported similar patterns, where patients with XP often present with severe disease at a late stage due to prolonged sun exposure without diagnosis or adequate photoprotection. This underscores the need for increased clinical vigilance, education of healthcare workers on early XP signs, and improved access to diagnostic services to enable timely diagnosis and intervention, which are critical to reducing morbidity and mortality in XP patients^[13–15]^.

XP has a 1000-fold greater incidence of skin malignancies than the general population does and a 50-fold greater risk of other systemic malignancies. Compared with the general population, which has an average age of 60 years, the mean age at presentation is 8 years. According to several case series, 40–60% of cutaneous and ocular neoplasms are malignant. Among these carcinomas, SCC affecting the face, head, and neck is the most common^[16,17]^, and the same has been reported in a study performed in Nepal between 2008 and 2012^[18]^. Due to their rapid growth, early metastasis, and potential for death, early identification of these cancers is crucial^[19]^. Therefore, metastatic malignant melanoma and SCC, which are caused by UV-induced skin hypersensitivity, are the main causes of mortality in XP patients^[20]^. The presence of all three forms of cancer in one patient is very uncommon; only a few cases have been documented in the literature where a patient presents with any two of these malignancies^[21]^. Due to the development of tumors, most untreated patients die before the age of 20 years^[1,22]^. Although early protection from UV exposure should be recommended, cutaneous neoplasms in XP individuals cannot be prevented^[20]^. Children with XP develop malignancies at a very young age, similar to our patient, and significant tissue damage has occurred by the time of diagnosis. In addition to sunburn, lentiginosis – a noticeable freckle-like pigmentation of the face – is typically the first manifestation discovered in patients^[1]^. It is first observed at approximately 2 years of age, as it was in our patient, and gradually settles in areas of the face that are exposed to the sun. In addition to photoaging, xerosis, skin laxity, and poikiloderma (dyspigmentation), the number of lentigines increases with time^[7]^. In addition to hemorrhagic crusted plaques and nodules across the left lower lid, our patient had many hypo- and hyperpigmented lesions throughout his face. Ocular abnormalities such as photophobia and keratitis, caused by UV-induced DNA changes to conjunctival, corneal, and eyelid epithelial cells, are common in people with XP^[7]^, but our patient had none of those complaints. The patient’s parents were heterozygous carriers of the relevant mutation and were cousins. The importance of preventing more consanguineous marriages should be emphasized in genetic counseling. XP results from a mutation in the excision nucleotide repair genes XPA to XPG and XPV. These genes encode proteins that are involved in repairing UV-induced photoproducts in DNA via NER, which is capable of repairing DNA damage caused by UV radiation^[3,23]^.

In addition to lesion biopsy samples that validate the suspicion of neoplasia and enable prompt excision of the neoformation, XP is diagnosed clinically^[24]^. There are currently no imaging or laboratory tests to confirm the diagnosis; instead, they are used to manage disease progression. The methods – sequentially genetic and unscheduled DNA synthesis techniques – that enable precise illness identification are still unavailable^[25]^.

## Conclusion

XP is an uncommon, autosomal recessive disorder with mutations in NER genes. Patients with XP are more likely to develop cutaneous and ocular neoplasms early in life. They are more aggressive and have a high recurrence rate. Thus, with follow-up, multimodal therapy is more beneficial than surgical treatment alone. There is no known treatment for XP; however, the quality of life and life expectancy of those who are affected can be significantly improved with greater awareness, early diagnosis, strict protection from sunlight, and careful patient management.

## Data Availability

Not applicable.
